# The Biological Potential Hidden in Inclusion Bodies

**DOI:** 10.3390/pharmaceutics12020157

**Published:** 2020-02-15

**Authors:** Laia Gifre-Renom, Joaquin Seras-Franzoso, Diana Rafael, Fernanda Andrade, Olivia Cano-Garrido, Francesc Martinez-Trucharte, Estefania Ugarte-Berzal, Erik Martens, Lise Boon, Antonio Villaverde, Ghislain Opdenakker, Simó Schwartz, Anna Arís, Elena Garcia-Fruitós

**Affiliations:** 1 Department of Ruminant Production, Institut de Recerca i Tecnologia Agroalimentàries (IRTA), 08140 Caldes de Montbui, Spain; laia.gifre@irta.cat; 2 Drug Delivery & Targeting, CIBBIM-Nanomedicine, Vall d’Hebron Institut of Research (VHIR), Universitat Autònoma de Barcelona (UAB), 08035 Barcelona, Spain; joaquin.seras@vhir.org (J.S.-F.); diana.fernandes_de_so@vhir.org (D.R.); fernanda.silva@vhir.org (F.A.); francesc.martinez@vhir.org (F.M.-T.);; 3 Networking Research Center on Bioengineering, Biomaterials and Nanomedicine (CIBER-BBN), 08035 Barcelona, Spain; olivia.cano.garrido@gmail.com (O.C.-G.); antoni.villaverde@uab.cat (A.V.); 4 i3S-Instituto de Investigação e Inovação em Saúde, Universidade do Porto, Rua Alfredo Allen 208, 4200-180 Porto, Portugal; 5 INEB-Instituto Nacional de Engenharia Biomédica, Universidade do Porto, Rua Alfredo Allen 208, 4200-180 Porto, Portugal; 6 Institut de Biotecnologia i de Biomedicina, Universitat Autònoma de Barcelona, 08193 Cerdanyola del Vallès, Spain; 7 Departament de Genètica i de Microbiologia, Universitat Autònoma de Barcelona, 08193 Cerdanyola del Vallès, Spain; 8 Laboratory of Immunobiology, Department of Microbiology, Immunology and Transplantation, Rega Institute for Medical Research, KU Leuven, University of Leuven, 3000 Leuven, Belgium; estefania.ugarteberzal@kuleuven.be (E.U.-B.); erik.martens@kuleuven.be (E.M.); lise.boon@kuleuven.be (L.B.); ghislain.opdenakker@kuleuven.be (G.O.)

**Keywords:** inclusion body, polymeric micelle, stability, matrix metalloproteinase-9, in vitro, in vivo

## Abstract

Inclusion bodies (IBs) are protein nanoclusters obtained during recombinant protein production processes, and several studies have demonstrated their potential as biomaterials for therapeutic protein delivery. Nevertheless, IBs have been, so far, exclusively sifted by their biological activity in vitro to be considered in further protein-based treatments in vivo. Matrix metalloproteinase-9 (MMP-9) protein, which has an important role facilitating the migration of immune cells, was used as model protein. The MMP-9 IBs were compared with their soluble counterpart and with MMP-9 encapsulated in polymeric-based micelles (PM) through ionic and covalent binding. The soluble MMP-9 and the MMP-9-ionic PM showed the highest activity values in vitro. IBs showed the lowest activity values in vitro, but the specific activity evolution in 50% bovine serum at room temperature proved that they were the most stable format. The data obtained with the use of an air-pouch mouse model showed that MMP-9 IBs presented the highest in vivo activity compared to the soluble MMP-9, which was associated only to a low and a transitory peak of activity. These results demonstrated that the in vivo performance is the addition of many parameters that did not always correlate with the in vitro behavior of the protein of interest, becoming especially relevant at evaluating the potential of IBs as a protein-based nanomaterial for therapeutic purposes.

## 1. Introduction

Inclusion bodies (IBs) are protein-based nanomaterials naturally formed under recombinant protein production processes. Although they have long been considered to be residual by-products of such processes, during the last decade, it has been extensively demonstrated that they are protein-based nanoparticles with a huge potential in the biotechnological and biomedical context [1,2]. Briefly, they have been described as a tunable and multi-effector material used as cell culture substrate for tissue-engineering purposes [3,4,5,6]. Their application as nanopills for protein-based cell therapies has also been proven in vitro [7,8]. In vivo, Unzueta and co-authors observed that subcutaneously injected tumor-targeted IBs could release the forming protein, cross into the blood stream and accumulate in the tumor for sustained periods [9]. In addition, when intratumorally injected, IBs made of therapeutic proteins, such as Omomyc and p31, induced tumor cells’ death [10], while cytokine-based IBs administered intraperitoneally conferred protection to zebrafish model from a lethal bacterial infection [11].

In most cases, IBs have been produced by using *Escherichia coli* as recombinant cell factory; however, their production has also been successfully done in lipopolysaccharide (LPS)-free recombinant systems, such as *Lactococcus lactis* [12,13].

Despite the large number of studies related to IBs’ applications, their in vitro and in vivo performances have never been compared with other protein-delivery formats, to determine if protein activity is the only factor to consider in protein-based treatments. For that, we have chosen matrix metalloproteinase-9 (MMP-9) protein as a model protein, as it has an important role degrading the extracellular matrix (ECM) in contexts of tissue development, involution and remodeling, and facilitating the migration of immune cells to the site of injury or inflammation [14,15]. Therefore, in the present work, we compared in vitro and in vivo performances of our model protein when displayed as IBs versus their soluble counterpart and an alternative nanocarrier, consisting in polymeric micelles (PM) loaded with MMP-9. The results showed that stability, format, and slow-release properties are parameters that should be considered, even more than biological activity, to design a therapeutic biomaterial with in vivo effects.

## 2. Materials and Methods

### 2.1. Bacteria Strains and Plasmids

*Lactococcus lactis* subsp. *cremoris* NZ9000 *clpP^−^ htrA^−^* (clpP-htrA; Em^®^, Jouy-en-Josas, France) strain [16,17] (kindly provided by INRA, Jouy-en-Josas, France; patent no. EP1141337B1) was used as the expression system for the production of the recombinant MMP-9 fragment Phe107-Pro449 (NCBI, NM_174744.2). Briefly, the gene was cloned into the pNZ8148 plasmid (Cm^®^, MoBiTec GmbH, Göttingen, Germany and transformed into competent *L. lactis* ClpP^−^ HtrA^−^ bacteria, as described elsewhere [18]. *L. lactis* clpP-htrA^−^ strain, containing a pNZ8148 plasmid cloned with a catalytically inactive form of MMP-9 (mutMMP-9; E402Q substitution [19,20]) was used as a control

### 2.2. Protein Production in L. lactis

Bacteria were grown overnight (O/N), at 30 °C, without shaking, in M17 broth supplemented with 0.5% glucose (M17G), 5 μg/mL of chloramphenicol, and 2.5 μg/mL of erythromycin. Cultures were re-inoculated at a 0.05 initial OD_660nm_ and induced with 12.5 ng/mL of nisin when OD_660nm_ achieved 0.4–0.6. Productions were sustained along 3 h, after which cultures were centrifuged at 6000× *g* for 30 min, at 4 °C, to recover bacteria. Pellets were frozen at −80 °C, until use.

### 2.3. Production and Purification of Soluble MMP-9

Soluble MMP-9 was obtained from the solubilization of MMP-9 IBs produced in *L. lactis*, as previously described [12]. Briefly, bacteria were suspended (30:500 PBS:culture, volume) and disrupted for 4 rounds by French press, at 1500 psi, ice-coated, and with protease inhibitors (Complete EDTA-free, Roche), followed by a 2 h incubation in 0.05 mg/mL of lysozyme, at 37 °C, in agitation. Lysates were pelleted (15,000× *g* for 45 min at 4 °C) and washed twice in mQ-H_2_O, and pellets were incubated in solubilization buffer (40 mM Tris pH = 8, 0.2% N-lauroyl sarcosine; 40:1 buffer:pellet, mL:g) for 40 h, at room temperature (RT). The supernatant was recovered at 15,000× *g* for 45 min, at 4 °C, filtered, and purified through Immobilized Metal Affinity Chromatography (IMAC), using a 1 mL HisTrap column (GE Healthcare, Chicago, IL, USA), in an ÄKTA purifier FPLC system (GE Healthcare, Chicago, IL, USA). Binding and elution buffers consisted in 20 mM Tris pH = 8, 500 mM NaCl, 20 or 500 mM Imidazole, respectively, and 0.2% N-lauroyl sarcosine. Four MMP-9 peaks were registered by holding the Imidazole gradient when elution absorbance (mAU) increased (data not shown), and they were dialyzed separately, O/N, in PBS at, 4 °C, in a stirrer. Possible precipitation was excluded (15,000× *g* for 15 min at 4 °C), and the purified soluble MMP-9 was aliquoted and stored at −80 °C, until use. The protein eluted in the first peak was selected for this study as the soluble MMP-9. The MMP-9 (MW: 39 kDa and *ε*: 70,080 M^−1^ cm^−1^; ProtParam-ExPASy, https://web.expasy.org/protparam/) in the samples was quantified by Nanodrop (Thermo Scientific, Waltham, MA, USA). The MMP-9 purity was analyzed by Coomassie blue staining. Briefly, the solubilized proteins were resuspended with Laemmli loading buffer (100 mM Tris, 8% glycerol, 55 mM SDS, 4% *β*-mercaptoethanol, and 1.6 M urea) and boiled for 5 min, and these were loaded into a 15% denaturing sodium dodecyl sulphate polyacrylamide gel. After electrophoresis, proteins in the gel were stained with Coomassie blue, and purity was determined for MMP-9 (ImageJ, version 1.46r).

### 2.4. Purification of MMP-9 and mutMMP-9 IBs

MMP-9 IBs and mutMMP-9 IBs were recovered and purified, as previously described [18]. Briefly, bacteria were suspended (30:50 PBS:culture, volume) and disrupted by French press—following above details—for 3 rounds, freeze/thawed (F/T), and agitated for 2 h, in 0.01 mg/mL of lysozyme, at 37 °C. After a new F/T, the solution was incubated at RT in 4 μg/mL of Triton X-100, for 1 h, in orbital agitation (OA), and aliquots were incubated O/N at 30 °C, in agar-M17G, intercalating F/T cycles until no bacteria colonies were grown. Afterward, the solution was incubated with 0.25 μL of NP-40 per sample mL (μL/mL) for 1 h, at 4 °C, and OA, and with 0.6 μL/mL MgSO_4_ (stock 1 M) and 0.6 μL/mL DNAse I (stock 1 mg/mL) for 1 h, at 37 °C, and OA. Pellets were obtained (6000× *g* for 30 min at 4 °C) and suspended in 5 mL of lysis buffer with 0.5% Triton X-100 per each 50 mL culture, F/T, pelleted again, and suspended in Dulbecco’s Phosphate-Buffered Saline (DPBS, GIBCO, Gaithersburg, MD, USA). MMP-9 IB pellet aliquots were obtained (20,000× *g* for 15 min) and stored at −80 °C, until use. The MMP-9 IBs quantification and purity were determined by Coomassie blue staining, following the procedure detailed for the solubilized MMP-9 in the previous section. In the case of IBs, samples were boiled for 40 min, preceding electrophoresis. Quantifications were performed by interpolation to a standard curve of solubilized MMP-9.

### 2.5. Polymeric Micelles (PM) Synthesis

The purified soluble MMP-9 was loaded in PM formed by the amphiphilic polymer Pluronic^®^ F127 (Kindly provided by BASF, Ludwigshafen am Rhein, Germany). Two different strategies were employed: (1) the encapsulation of MMP-9 by electrostatic interaction with the polymer and (2) the covalent binding of the protein to a previously activated F127-COOH (synthesized as previously described [21]). In both cases, PM were prepared by using the film hydration procedure. Briefly, 20 mg of F127 polymer for strategy (1) and a mixture (8:2, *w*/*w*) of F127:F127-COOH for strategy (2) were weighted and dissolved in a methanol:ethanol (1:1, *v*/*v*) solution. Solvent was removed by evaporation under vacuum conditions for 10 min, at 60 °C, and 200 rpm in a rotary evaporator IKA RV10; the film was further air-dried O/N at RT. The film was hydrated in 2 mL 20 mM Tris-HCl containing MMP-9 at 1 mg/mL for MMP-9 by 5 min of vortex at RT, for protein encapsulation through electrostatic interactions. Self-assembled PMs were further dialyzed against 20 mM Tris-HCl in Float-A-Lyzer^®^G2 Dialysis devices with a 100 KD pore size (Repligen Europe B.V., Breda, The Netherlands). For covalent binding of MMP-9 F127:F127-COOH films were hydrated in 1 mL 20 mM Tris-HCl, as described above, and incubated with 1-etil-3-(3-dimethylaminopropyl) carbodiimide (EDC) (Sigma-Aldrich, Merck Life Science S.L.U., Madrid, Spain) at 10 µg/mL for 30 min at RT, under vigorous agitation. Then 1 mL of MMP-9 solution at 2 mg/mL was added and further incubated for 2 h at RT, under agitation. Finally, PM were dialyzed following the same procedure that was employed for PMs encapsulating MMP-9.

### 2.6. Transmission Electron Microscopy (TEM) Imaging

The TEM images of MMP-9 IBs were obtained, as previously described [18]. For PMs’ images, a 6 µL drop, 1:20 dilution, was gently placed over 400 mesh cupper grid, previously coated with carbon. Samples were incubated for 1 min at RT, and liquid excess was blotted with Whatman No.1 filter paper. Negative staining was performed by the addition of a 6 µL drop of uranyl acetate 2% (*w*/*v*), 1 min at RT. Staining excess was removed as described above. PMs observation was carried out in a TEM Jeol JEM-1400 (Jeol Ltd., Croissy-sur-Seine, France) operated at 120 kV. Images were further processed, using the software ImageJ NIH (version 1.45s).

### 2.7. MMP-9 Loading Efficacy

MMP-9 loading efficacy in PM was determined by the comparison of the protein amount in the samples before and after the dialysis process described in the Polymeric Micelles Synthesis section. Protein amount was measured by bicinchoninic Acid (BCA) method, following manufacturer’s indications (Pierce). Plates were measured at 590 nm in an ELx800™ microplate reader (BioTek™, Winooski, VT, USA).

### 2.8. X-ray Photoelectron Spectroscopy (XPS)

XPS measurements were performed at room temperature with a SPECS PHOIBOS 150 hemispherical analyzer (SPECS GmbH, Berlin, Germany), in a base pressure of 5 × 10^−10^ mbar, using monochromatic Al Kalpha radiation (1486.74 eV) as excitation source.

### 2.9. Protein Stability Assay

Soluble MMP-9, the ionically bound and covalently bound MMP-9 nanopolymers, and the MMP-9 IBs were incubated at 0.2 mg/mL in 50% bovine serum or PBS at RT and samples were analyzed for the MMP-9 capability to degrade a dye-quenched gelatin (DQ gelatin™, Invitrogen, Carlsbad, CA, USA). Because the initial velocity of the gelatin catalysis by the MMP-9 IBs was lower than by the soluble or encapsulated MMP-9 (data not shown), after 0 h, 2 h, 8 h, 24 h, 48 h, 72 h, 7 days, and 14 days of incubation, 100 or 10 µg of MMP-9 IBs or solubilized and encapsulated MMP-9, respectively, were plated in a black 96-well plate with transparent flat-bottom containing assay buffer (5 mM CaCl_2_, 50 mM Tris pH 7.6, 150 mM NaCl, and 0.01% Tween20). Immediately before reading the plate, 0.25 µg of DQ-gelatin was added to the wells, and the plate was bottom-read at a 495/515 excitation-emission wavelength in a Victor III multilabel counter (Perkin-Elmer, Waltham, MA, USA), with repeated reads every 2.3 min, along 1 h. For each kinetic, the specific activity was obtained through the initial velocity calculation (rfu/min) corrected by the MMP-9 µg in the respective well (rfu/min/µg), and the % of specific activity was obtained for each sampling time, using the time 0 h in PBS as the reference.

### 2.10. In Vivo Comparison of the Inflammatory Response to Solubilized MMP-9 versus MMP-9 IBs, Using a Mouse Model

Air pouches were provoked on the back of sixty-nine C57BL/6 MMP-9 KO mice [22] by injecting 3 mL of filtered air intra-dermally on days 0 and 3, as detailed in Vandooren et al. [23]. Then, 40 µg of solubilized MMP-9 or MMP-9 IBs was injected into the pouches at day 6 in 200 µL of DPBS, and air-pouch exudates were collected after 3, 24, and 48 h after the protein injections. Mice were euthanized with 40 mg/kg of Dolethal (pentobarbital) intraperitoneally, and the air-pouch exudates were obtained by injecting, massaging, and recovering 2 mL of PBS with heparin 20 U/mL, and repeating the process without taking out the needle with 3 more mL. All experimental procedures were approved by the institutional Ethics Committee (KU Leuven), under license LA1210243, for animal welfare (Project 277/2014, 1 January 2016). Exudates were cooled on ice, and viable cells were counted with a Neubauer chamber. About 50 to 80 × 10^3^ cells per sample were centrifuged on microscope slides, using a Shandon Cytospin 2 centrifuge (Thermo Shandon, Pittsburgh, PA, USA), at 75× *g* for 10 min, and preparations were fixed and stained with hematoxylin–eosin and analyzed under a light microscope for immune cell populations.

### 2.11. Statistical Analysis

For the stability assay, samples were incubated per duplicate. For the in vivo assay, 12 exudates were analyzed after 3 h of protein injections, 24 after 24 h, and 24 after 48 h. Variables were transformed to normalize data when necessary, and data were analyzed by using the fixed effect models (JMP, SAS Institute Inc., Cary, NC, USA). For the stability assay, format or incubation time was used as a fixed effect when appropriate. For the in vivo assay, treatment, exudate-collecting time, and their interaction were used as fixed effects. Animals were not considered a random effect, as no repeated measures were performed. Non-transformed means are represented in all graphs. Non-transformed SEM are represented in the stability assay graphs, while transformed SEM are represented in the in vivo assay graphs. Moreover, *p*-values and letters correspond to the Tukey test analyses, using transformed data when required.

## 3. Results

### 3.1. Activity of MMP-9 Nanoparticles

**Figure 1:**
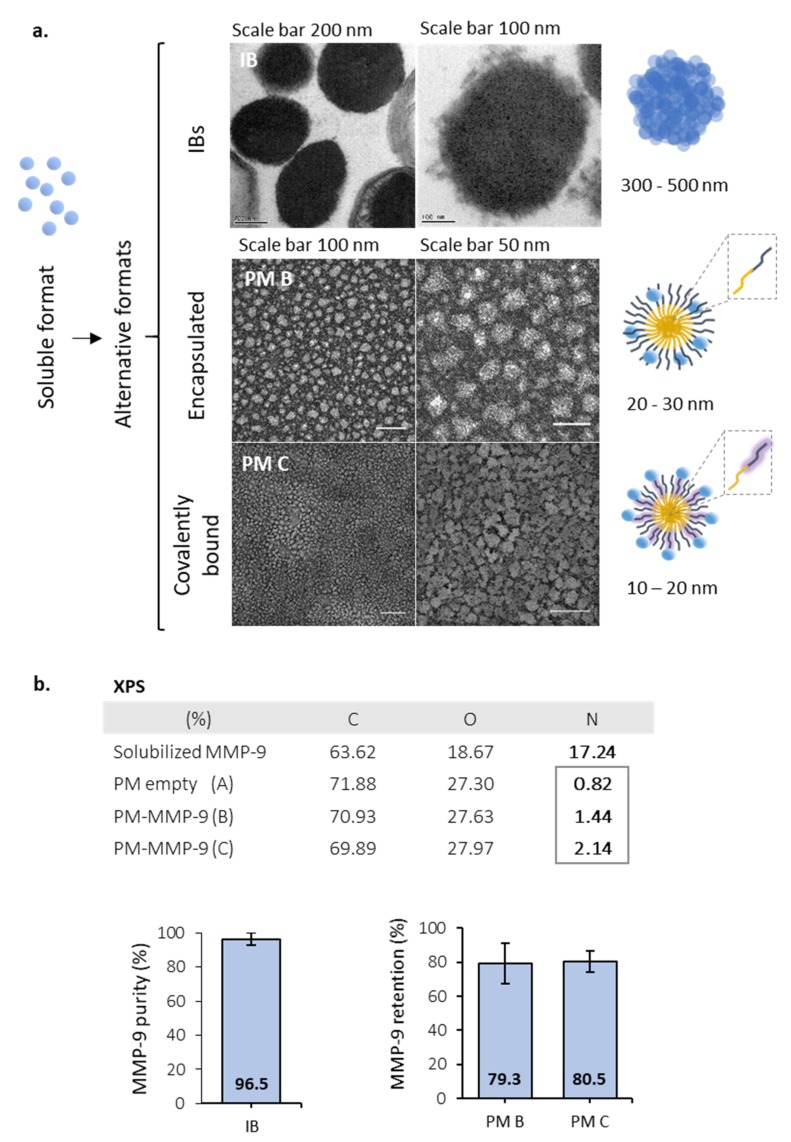
Description for the MMP-9 formats. (**a**) Transmission electron microscopy (TEM) images for MMP-9 IBs (IB), non-covalent MMP-9-nanopolymers (PM B), and covalent MMP-9-nanopolymers (PM C). Pictures represent the format structures for the soluble proteins, IBs, and Pluronic^®^ F127 amphiphilic polymers. Magnified, F127 hydrophobic chains are represented in yellow, and hydrophilic chains in dark blue. In PM C, the carboxylated chains of polymer (F127:COOH) are represented in dark blue within a pruple shadow and correspond to 25% of the total polymer (ratio 1:4). Soluble MMP-9 are represented as single blue spheres. (**b**) XPS results summary showing elemental composition on the surface of MMP-9, non-loaded PM (A), and MMP-9 loaded PM by the two strategies previously mentioned (PM B and PM C). Panels below, on the left MMP-9 purity of IBs. On the right, protein encapsulation efficacy for both types of PM tested. Bars depict SE.

MMP-9 was successfully produced in *L. lactis* in its soluble form and as bacterial IBs, rendering nanoparticles in the range of 300 to 500 nm in diameter (Figure 1a). In a second step, soluble MMP-9 (97.4% purity) was loaded in polymeric micelles (PM), using two different approaches: encapsulated by electrostatic interactions (named PM B) and covalently bound (named PM C) (Figure 1a). PM B and PM C, as it occurs with IBs, were pseudo-spherical nanoparticles with sizes of 20–30 and 10–20 nm, respectively (Figure 1a). Besides, as shown by the slight increase in N presence in PM B and PM C in comparison to empty PM (XPS table, Figure 1b), a certain amount of protein (major source of N) was effectively bound to the PM surface for both protein loading strategies. Analyzing MMP-9 cargo in further detail, we observed the encapsulation efficiency of MMP-9 within the polymer was around 80%, being similar in covalent and ionic PM (Figure 1b). The percentage of MMP-9 forming IB nanoparticles achieved values of 96.5% (Figure 1b).

**Figure 2:**
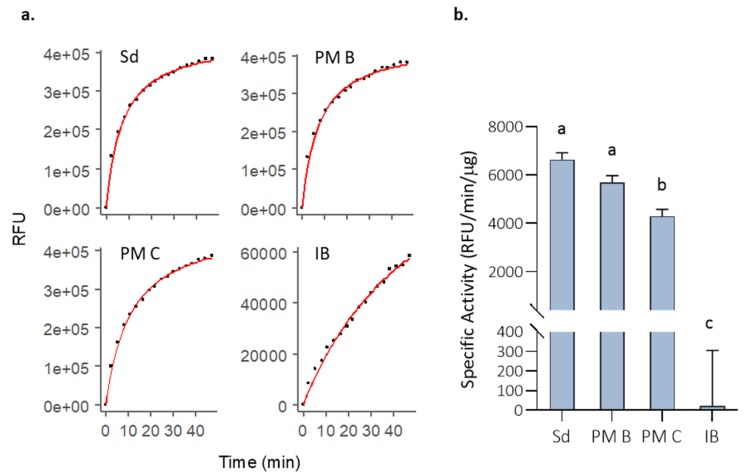
Initial MMP-9 activity (*t* = 0 h) in PBS incubations for the analyzed formats. (**a**) DQ-gelatin degradation kinetics for one of the replicates of 10 µg of solubilized MMP-9 (Sd), 10 µg of non-covalent MMP-9-PM (PM B), 10 µg of covalent MMP-9-PM (PM C), and 100 µg MMP-9 IBs (IB). Fluorescence reads along time (dots), and R model curves (red line) are represented. RFU = Relative fluorescence units. (**b**) Comparison of the specific activity (RFU corrected by time and MMP-9 µg) for the analyzed formats. Means and SEM are represented. Data were analyzed, using a fixed effect model with format as fixed effect. Different letters depict significant differences (*p* = 0.0003).

The activity of MMP-9 in the different nanostructured formats was tested in vitro and compared with the soluble (non-nanoclustered) form (Figure 2). Although all forms were functional, we observed that the free MMP-9 and the MMP-9 encapsulated in PM B nanoparticles showed the highest activity values (Figure 2b). Polymeric micelles with covalently bound MMP-9 (PM C) showed a decrease of 10% in the specific activity (Figure 2). However, it is especially relevant to underline the low activity values of IBs, which had an activity 260 and 196 times lower than PM B and PM C, respectively (Figure 2b).

### 3.2. In Vitro MMP-9 Stability

**Figure 3:**
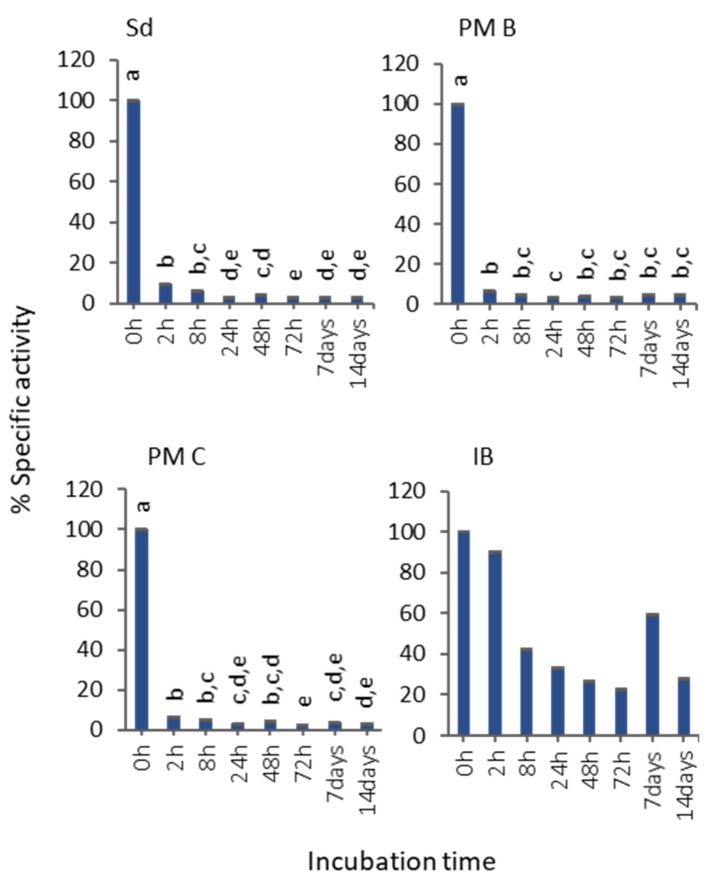
Evolution of the MMP-9 activity at different time points of incubation in serum 50% (*v*/*v*). Non-transformed means and transformed SEM are represented. Data were analyzed, using a fixed effect model, with format as a fixed effect. Different letters depict significant differences: solubilized MMP-9 (Sd), *p* < 0.0001; non-covalent MMP-9-nanopolymers (PM B), *p* = 0.0023; covalent MMP-9-nanopolymers (PM C), *p* < 0.0001; MMP-9 IBs (IB), *p* = 0.84.

Although IBs showed the lowest activity values (Figure 2b), the specific activity evolution in 50% bovine serum at room temperature proved that they were the most stable format (Figure 3). The assessment of specific activity decrease revealed that soluble protein and polymeric nanoparticles lost almost all the activity after 2 h of incubation, while IBs kept a 28% of the activity after 14 d of incubation (Figure 3).

### 3.3. In Vivo MMP-9 Activity

**Figure 4:**
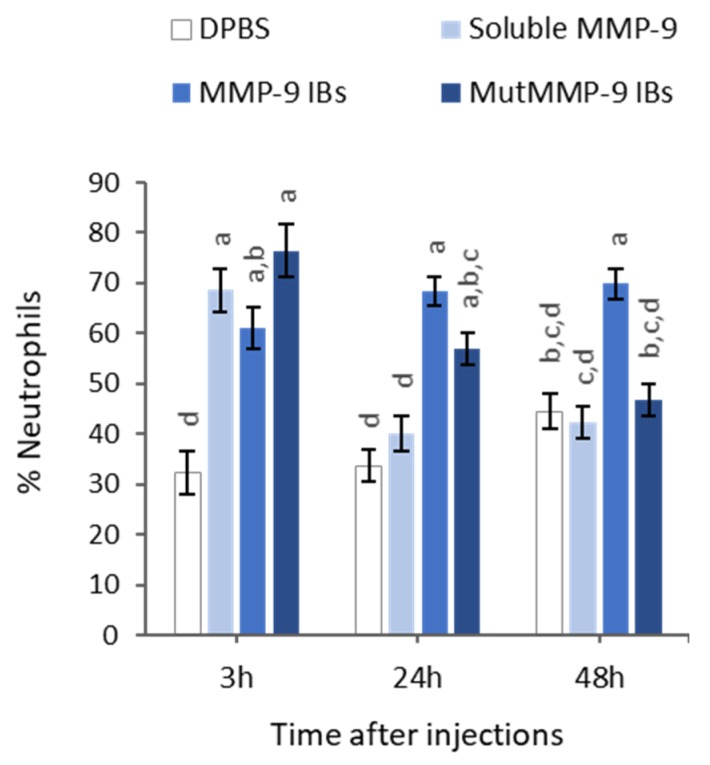
Relative quantification of neutrophils among total immune cell populations by cytospin centrifugation of exudates recovered after 3, 24, and 48 h after intradermal injection of Sb MMP-9, MMP-9 IBs and mutant MMP-9 IBs in air-pouches of *mmp9* KO mice. Percentages are relative to 100 cells counted by triplicate for each sample. Means and SEM from non-transformed data are represented. Different letters depict significant differences (*p* < 0.0001). Data were analyzed, using a fixed effect model with treatment and time as fixed effects (*n* = 12 (3 h), *n* = 24 (24 h), *n* = 24 (48 h)).

With the aim to determine if the results observed in vitro with MMP-9 IBs correlated with their performance in vivo, the soluble MMP-9, showing the highest biological activity (Figure 2), and MMP-9 IBs, showing the lowest activity but highest stability, were compared in *mmp9* knock-out mice [22], following an air-pouch experimental model [23]. Mutant MMP-9 IBs, which are non-functional MMP-9 IBs formed by an enzymatically inactive mutant form of MMP-9, were also administered, to differentiate the specific MMP-9 effect from other components present in IBs but different from the recombinant MMP-9. After injections with DPBS (negative control), soluble MMP-9, MMP-9 IBs (active IBs), or mutMMP-9 IBs (inactive IBs), in mouse air-pouches, exudates were recovered and centrifuged at three different time points (3, 24, and 48 h), to analyze the number of neutrophils that migrated to the site of injection due to the action of MMP-9 at degrading the ECM. At 3 h post-injections, there were no significant differences in neutrophils percentage for any of the MMP-9 treatments (soluble MMP-9, MMP-9 IBs, and mutMMP-9 IBs) (Figure 4). However, cell recruitment at 24 h was higher in those animals injected with both MMP-9 IBs and mutMMP-9 IBs in comparison with the DPBS control and the soluble MMP-9 (Figure 4). While neutrophil count values of those animals treated with mutMMP-9 IBs returned to basal levels at 48 h, the total cell recruitment levels were kept high in those exudates of animals injected with active MMP-9 IBs (Figure 4).

## 4. Discussion

Many examples have been published during the last decade that prove that IBs are protein-based biomaterials with promising characteristics to be used for cell replacement therapies, for tissue engineering purposes, or even for cancer treatment [3,4,5,6,7,8,9,10,11,24], among other applications. However, their characteristics and their in vitro behavior have never been correlated with their in vivo performance compared with other standard formats. For that, we have evaluated the functionality and stability of a protein with relevance in different fields, such as MMP-9 produced in distinct formats: (i) soluble (naked) form; (ii) encapsulated in PM by ionic interactions or covalently bound to the PM surfaces (PM B and PM C nanoparticles, respectively); and (iii) IBs nanoparticles (Figure 1). Activity values bring out two important messages: MMP-9 IBs’ functionality is clearly poorer than MMP-9 in its soluble form or as polymeric nanoparticles, showing values between 200 and 250 times lower (Figure 2); and nano structuring of MMP-9 as PM B and PM C did not improve the functionality of our model protein (Figure 2). In fact, we observed a slight decrease in the MMP-9 activity encapsulated in PM C (Figure 2), probably due to the encapsulation process, which can negatively affect protein activity. On the other hand, the stability analysis of MMP-9 in the different forms evaluated revealed that IBs are much more stable than soluble and MMP-9 PM B and PM C nanoparticles (Figure 3). IBs keep an important part of their initial activity after 14 d, which proves that these protein nanoparticles confer protection to the embedded protein. Overall, in line with the findings set out above, it becomes clear that, in terms of activity, the soluble version of MMP-9 is the best option (Figure 2), while the most stable format is IBs (Figure 3). Based on these results, we compared the in vivo response of mice treated with soluble MMP-9 and MMP-9 IBs, to determine if the difference in stability shown by IBs could have relevance in a real context where proteases and other elements of a harsh environment are present. The data obtained by using an air-pouch mouse model demonstrate that the in vivo performance is the result of many parameters that do not always correlate with the in vitro behavior of the evaluated protein (Figure 4). Clearly, at the longest treatment time, only those animals injected with IBs kept neutrophil levels higher than those treated with DPBS, being relevant that, at 48 h, the neutrophil proportion after MMP-9 IBs treatment was significantly higher than for mutMMP-9 IBs, which are formed by an MMP-9 inactive form (Figure 4). The residual effect of mutMMP-9 indicates that there is a transitory and basal inflammatory effect due to an unspecific effect of protein nanocluster format, but the results distinguished clearly this background from the effect of heterologous protein embedded in the IBs. In addition, the effect of soluble MMP-9 was restricted to the first 3 h and disappeared at longer times, indicating that, despite the high activity of MMP-9 observed in vitro, it is not translated to any relevant effect in an in vivo context.

Thus, as it has previously proven, IBs have great potential, being in many cases totally or partially hidden when their action is evaluated in vitro. The results presented here prove that, to observe a desired effect of the administered protein in vivo, it is not only their biological activity that is important, but also other parameters, such as stability, format, and probably the slow-release properties that IBs have, and that have been previously described [1,25]. In consequence, IBs are not only a promising format because they are produced through a cost-effective one-step process [25], but also because low amounts of functional protein in this format are enough to render desired significant response in vivo.

## 5. Conclusions

Although the in vitro results showed that IBs have much lower activity than their soluble counterpart and other MMP-9-based nanoparticles, the in vivo mouse model indicated that this low in vitro activity is transformed in a relevant in vivo effect when administered to the animals. The results of this study showed that the evaluation of in vitro activity of IBs do not reflect their therapeutic potential, bringing to light the importance to integrate other parameters such as protein stability and the use of in vivo animal models when protein-based nanoclusters are evaluated as a therapeutic biomaterial.
